# Ethylene Acts as a Negative Regulator of the Stem-Bending Mechanism of Different Cut Snapdragon Cultivars

**DOI:** 10.3389/fpls.2021.745038

**Published:** 2021-09-30

**Authors:** Aung Htay Naing, May Thu Soe, Jeong Hyun Yeum, Chang Kil Kim

**Affiliations:** ^1^Department of Horticulture, Kyungpook National University, Daegu, South Korea; ^2^School of Biofibers and Biomaterials Science, Kyungpook National University, Daegu, South Korea

**Keywords:** ethylene production, ethephon, lignin content, stem curvature, silver thiosulfate

## Abstract

This study investigated whether ethylene is involved in the stem-bending mechanism of three different snapdragon cultivars ‘Asrit Red’, ‘Asrit Yellow’, and ‘Merryred Pink’, by treating their cut stems with an ethylene-releasing compound (ethephon), an ethylene-action inhibitor [silver thiosulfate (STS)], and distilled water (as the control). Ethephon completely prevented stem bending in all cultivars, whereas STS exhibited a higher bending rate compared with the control. The bending rates were influenced by several factors, such as the degree of stem curvature, relative shoot elongation, ethylene production, and lignin content, indicating their involvement in the stem-bending mechanism of the cultivars. The analysis of the expression of genes involved in the ethylene and lignin biosynthetic pathways also supported the importance of lignin and ethylene in the stem-bending mechanism. Taken together, as ethephon completely prevented stem bending of the three snapdragon cultivars, this study suggested that ethylene acts as a negative regulator of the stem-bending mechanism of snapdragon cultivars, and the information will be valuable for the prevention of stem bending in other commercially important ornamental flowers.

## Introduction

The vase life of cut snapdragon flowers (*Antirrhinum majus* L.) is usually determined by the stem-bending rate, rather than petal senescence, because they cannot be used for commercial purpose once their stems are bent. Unfortunately, stem bending often occurs in snapdragon cut flowers during a short vase-life period. Hence, stem bending is a major postharvest problem of cut snapdragon flowers. Research has been conducted using ET and ET-action inhibitors, such as silver thiosulfate (STS) and 1-methylcyclopropene (1-MCP), to investigate whether ET is involved as a positive regulator in the stem-bending mechanism of snapdragon (Philosoph-Hadas et al., 1996, 1999; Woltering et al., 2005; Çelikel et al., 2010). In a study reported by Çelikel et al. (2010), pretreatment of the stems with STS or 1-MCP did not significantly affect the gravitropic curvature and bending of snapdragon flowers compared with the control, while 1-MCP-treated stems even showed slightly increased bending compared with ET-treated stems (Woltering et al., 2005). However, in the studies reported by Philosoph-Hadas et al., (1996, 1999), STS or 1-MCP blocked the gravitropic response of the snapdragon flowers. According to the findings obtained in those studies, the role of ET in the stem-bending mechanism of snapdragon remains unclear. Therefore, further research (using ET-action inhibitors, ET-enhancers, and different plant genotypes) is still required to validate the role of ET in the stem-bending mechanism of cut snapdragon flowers.

Stem bending in cut flowers is generally caused by the presence of a low lignin content in the stems (Marousky, 1986; Naing et al., 2017), which leads to an inability to strengthen the rigidity of the stem and the vascular tissue (Steinitz, 1982, 1983). This will result in reduction of the mechanical resistance to the whole-flower spikes, as well as disruption of water and mineral transport to the flowers, which will in turn cause rapid stem bending. In cut tulip flowers, treatment of cut stems with the ET-releasing compound 2-chloroethylphosphonic acid (ethephon) controlled stem elongation that accelerated stem bending (Nichols, 1971; Moe, 1980). In addition, van Doorn et al. (2011) also reported that ethephon controlled stem elongation and delayed stem bending of cut tulip. This compound has been reported to improve resistance to stem breakage by maintaining lignin content and triggering lignin biosynthetic genes [phenylalanine ammonia-lyase (*PAL*) and 4-coumarate: CoA ligase (*4CL*)] encoding the first (PAL) and third (4CL) enzymes of lignin biosynthesis pathway (Brown, 1990; Hyodo et al., 1991; Ievinsh and Romanovskaya, 1991; Sitbon et al., 1999; Luo et al., 2007; Huang et al., 2013; Mangieri et al., 2016; Ye et al., 2016). However, to the best of our knowledge, the role of ethephon in the stem-bending mechanism of snapdragon flowers has not been well investigated. Therefore, it was quite interesting to investigate whether ethylene is involved as a negative regulator in the stem-bending mechanism of cut snapdragon flowers by treating the cut stems of three different cultivars with an ET-action inhibitor (STS) and an ET-enhancer (ethephon). In addition, it was also of interest to investigate the association between ET production and lignification in the stems of cut snapdragon flowers by measuring ET production, lignin content, and the levels of expression of genes (*PAL* and *4CL*) involved in the lignin biosynthetic pathways. The enzymes 1-aminocyclopropane-1-carboxylic acid (ACC), synthetase (ACS), and ACC oxidase (ACO) are mainly involved in the ET biosynthesis pathway (Yang and Hoffman, 1984). Therefore, the expression levels of *ACS* and *ACO* genes encoding the corresponding enzymes were also investigated in the cut stems of the snapdragon.

In this study, we treated the stems of three different snapdragon cultivars with an ET-enhancer (ethephon) and an ET-action inhibitor (STS) and investigated the involvement of ET in the stem-bending mechanism of these flowers by determining stem curvature, bending rate, ET production, and its related gene expression, as well as lignin content and its related gene expression.

## Materials and Methods

### Plant Material

The three different snapdragon cultivars ‘Asrit Red’, ‘Asrit Yellow’, and ‘Merryred Pink’ were cultivated in a farm located near Busan. Flowers with 4–6 open flowers per inflorescence and free of mechanical and disease defects were selected and harvested in the early morning. They were then immediately transported to the laboratory of Kyungpook National University in Daegu. Once arriving at the laboratory, the flower stems were trimmed to a length of 50cm and excessive leaves were gently removed. The cut flowers were then used to investigate the stem-bending mechanism.

### Treatment of Cut Flowers With STS, Ethephon, and Distilled Water (as a Control)

Ethephon, an ET-releasing plant regulator, not only regulates plant growth and flowering but also enhances lignification *via* the liberation of ET (Rajala et al., 2002; Haque et al., 2007; Mangieri et al., 2016; Ye et al., 2016), whereas STS blocks ET action by binding ET-receptor sites (Hoffman and Yang, 1982). The cut flowers were separately placed in bottles (1L) containing 0.2mm STS, 0.25mm 2-chloroethylphosphonic acid (ethephon; Sigma-Aldrich), or distilled water (as a control) for 24h. STS was prepared by adding 0.2mm silver nitrate (Sigma-Aldrich) to 1.2mm sodium thiosulfate (Sigma Aldrich). The range of concentrations for ethephon and STS was selected based on the work of van Doorn et al. (2011). The treated stems were then thoroughly washed under tap water and placed in new bottles containing only distilled water (500ml). We included three bottles per treatment, and each bottle contained ten flowers. The flowers were then placed in a controlled chamber setting at 20°C with a relative humidity of 70%, a light intensity of 15μmolm^−2^ s^−1^, and a 12-h photoperiod, which was similar to the condition used by Philosoph-Hadas et al. (1996).

### Assessment of Stem Curvature (Degree) and Bent Stems (% of Total)

The stem-curvature degree was measured using a protractor, and stems in which the upper part exhibited an angle >45° were recorded as bent stems. The curvature of all stems (*n*=30) was measured at 3, 6, and 9days after treatment (DAT). Each measurement included ten flower stems and was repeated three times. All bent stems were evaluated at 3, 6, and 9 DAT, and the results are reported as percentages.

### Determination of RSE

For the determination of Relative Shoot Elongation (RSE), the site of the stem located 100mm away from the apex of the inflorescence stem was marked with a felt pen before treatment with STS, ethephon, and distilled water. The growth of the flower stem was then monitored at 3, 6, and 9 DAT. Each measurement included ten flowers and each measurement was repeated three times.

### Analysis of Lignin Content

Lignin analysis was performed according to the method described by Syros et al. (2004). The parts from the bending zone of the treated stems were sampled at 3, 6, and 9 DAT. The samples were pulverized in 95% ethanol using a mortar and pestle, to obtain a homogeneous suspension. Subsequently, the homogenates were centrifuged at 1,000×*g* for 10min, and the pellets were washed with 95% ethanol, followed by a 95% ethanol-to-n-hexane solution (1:2v/v) three times. After drying, 20mg of the deposits were dissolved in 0.5ml of 25% acetyl bromide in glacial acetic acid for 30min at 70°C. After rapid cooling, 0.9ml of NaOH (M) was added, to stop the reaction, followed by the addition of 0.1ml of hydroxylamine-HCl (7.5M) and 5ml of glacial acetic acid. Subsequently, 0.1ml of the reaction mixture was diluted with 3ml of ice-cold acetic acid. After centrifugation at 1,000×*g* for 5min, the supernatant was measured at 280nm. Lignin content was expressed as a 280mg^−1^ protein. Three independent bending zones were assessed.

### Measurement of ET Production

Ethylene (ET) production was analyzed in the bending zone of stems treated with STS, ethephon, and control vehicle. The bending zones were sampled at 6 DAT. They weighed approximately 2–3g and were placed in 30-ml Erlenmeyer flasks, sealed with rubber serum caps, and incubated for 20h at room temperature for ET accumulation. ET production was measured using gas chromatography (7820 GC), and the ET-production rates were calculated and expressed as nl/g/h, as described by Xu et al. (2021). Three independent bending zones were used for each analysis, with three replicates.

### Analysis of the Expression of Genes Involved in the Lignin and ET Biosynthetic Pathways

Total RNA was extracted from 100mg of stem segments from the bending zone, which were sampled at 3, 6, and 9 DAT, using the RNeasy Plant Mini Kit (Qiagen, Hilden, Germany). cDNA was synthesized from 1μg of total RNA using an oligo dT20 primer and a reverse transcription kit (ReverTra Ace-á, Toyobo, Japan). The mRNA levels of lignin biosynthetic genes [*PAL* (DQ866660.1) and *4CL* (Y15607.1)] were analyzed using the StepOnePlus Real-Time PCR system (Thermo Fisher Scientific, Waltham, MA, United States). Similarly, the transcript levels of ethylene biosynthetic genes [*ACS1* (AF083814.2), *ACO1* (AY333925.1), and *ACO2* (AY333926.1)] were detected from the bending zones of stems sampled at 6 DAT. The primers and PCR conditions used for this analysis are listed in Supplementary Table 1. Three independent bending zones were used for each analysis, with three replicates.

### Statistical Analysis

Data were analyzed using SPSS v. 11.09 (IBM Corporation, Armonk, NY, United States) and are presented as the mean of three replicates. The least significant difference test (LSDT) and Duncan’s multiple range test (DMRT) were used to assess the differences between mean values. The significance level was set at *p*<0.05.

## Results

### Ethylene Negatively Regulates the Stem Curvature and Bending of Cut Flowers From Different Snapdragon Cultivars

**Figure 1:**
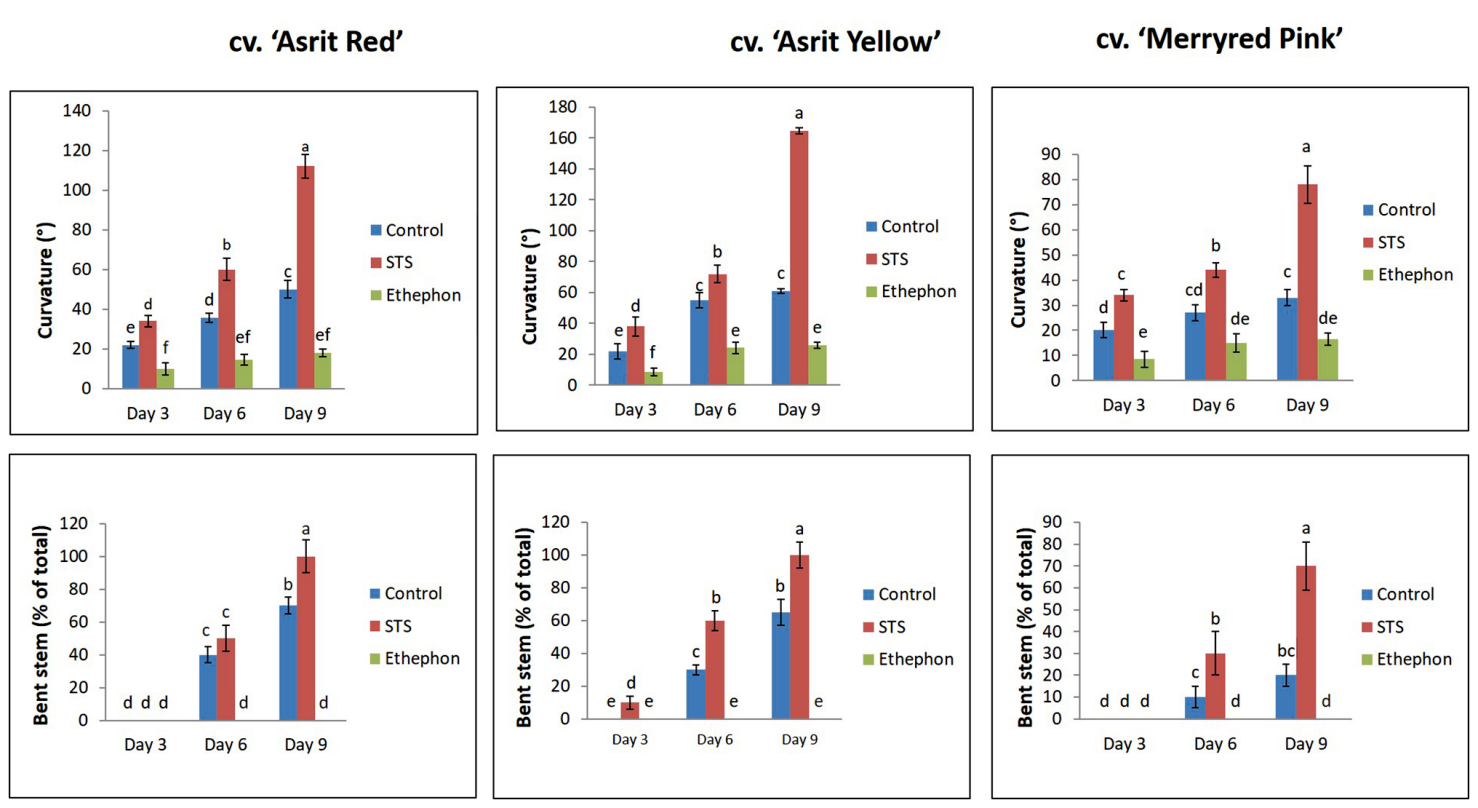
Comparison of the curvature degrees and bending rates of the stems of three different snapdragon cultivars at 3, 6, and 9 days after treatment (DAT) with silver thiosulfate (STS), ethephon, and control. The data represent the means of three replicates, and the error bars indicate the standard error. Means with the same letters are not significantly different by Duncan’s multiple range tests (DMRT, *p*<0.05).

The cut stems of flowers from three different snapdragon cultivars were treated with an ethylene-releasing compound (ethephon), an ethylene-inhibiting compound (STS), and distilled water (control). Varying degrees of stem curvature in response to the treatments were observed in all cultivars at 3 DAT, with the degree of stem curvature observed for the STS treatment being significantly higher than that of the control, and that observed in the ethephon-treated stems being the lowest of the three conditions (STS>control>ethephon). When increasing the vase life period from 3 to 6days, a significant increase in the stem curvature degree was observed in all cultivars treated with STS, in two cultivars with control treatment, and in one cultivar with ethephon treatment (Figure 1). A further significant increment in the curvature degrees in STS treatments and a slight increment in the curvature degrees in control treatments were observed when the vase life was extended to 9days; however, such increment was not observed in the ethephon treatments in all cultivars. Despite a slight variation in stem curvature among the cultivars in response to the treatments, ethephon significantly inhibited the induction of stem curvature in all cultivars, whereas STS strongly induced stem curvature, with the curvature induced by the STS treatment being significantly greater than that afforded by the control treatment in all cultivars at 3, 6, and 9 DAT, respectively.

**Figure 2:**
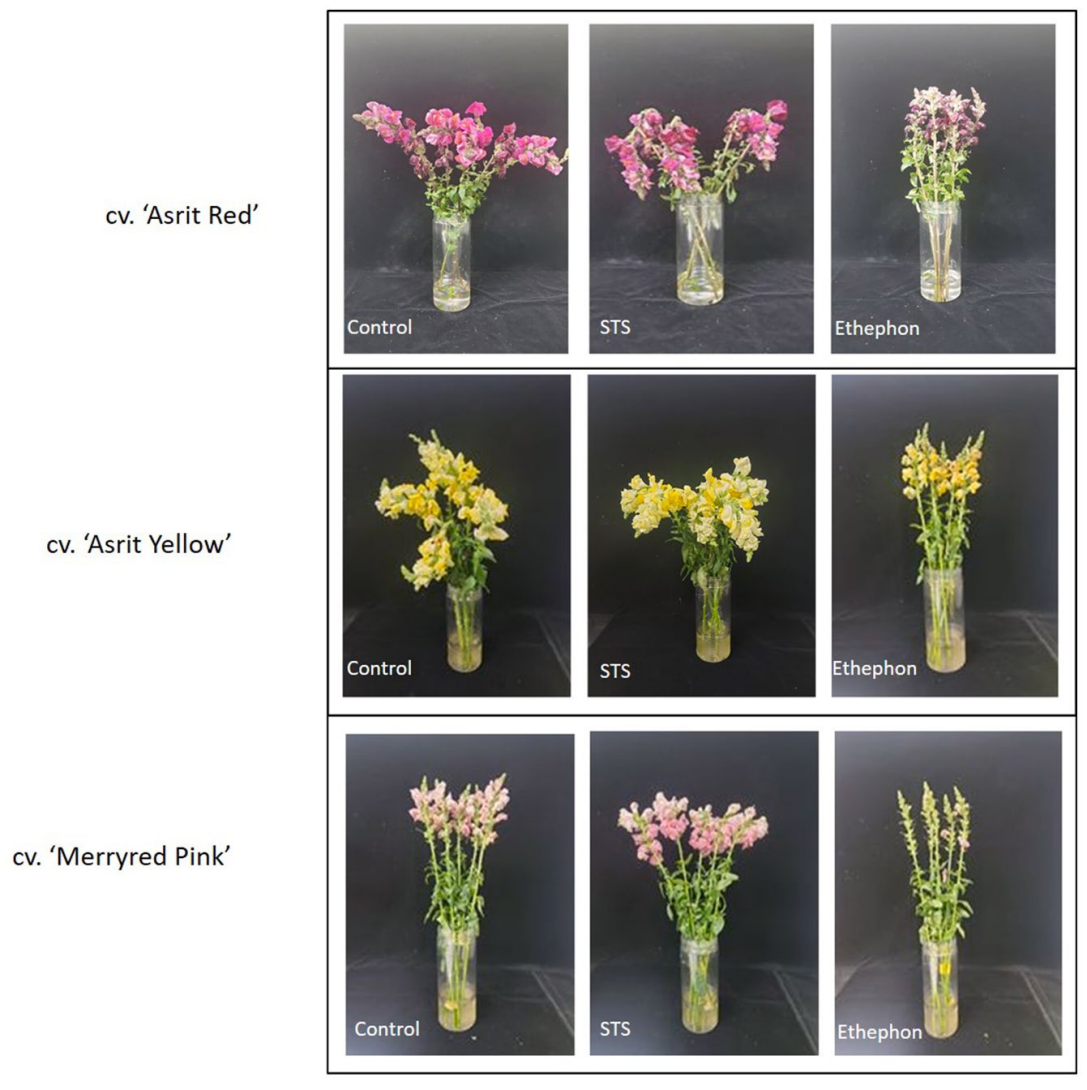
Comparison of the bending status of three different snapdragon cultivars treated with control vehicle, STS, and ethephon. These photographs were taken at 9 DAT.

The assessment of the stem-bending rate in all cultivars revealed no bending in all treatments at 3 DAT, except for the cv. ‘Asrit Yellow’ cultivar treated with STS, which exhibited a bending rate of 10%. At 6 DAT, stem bending was distinctly observed in all cultivars treated with STS and the control, whereas stems treated with STS exhibited bending rates of 60% in the ‘Asrit Yellow’, 50% in the ‘Asrit Red’, and 30% in the ‘Merryred Pink’ cultivars. In turn, treatment with the control exhibited bending rates of 30% in the ‘Asrit Yellow’, 40% in the ‘Asrit Red’, and 10% in the ‘Merryred Pink’ cultivars, respectively. At 9 DAT, the stem-bending rates were higher than those observed at 6 DAT in all cultivars, because the stems treated with STS exhibited bending rates of 100% in the cvs ‘Asrit Yellow’ and ‘Asrit Red’ cultivars and 70% in the cv. ‘Merryred Pink’, and those treated with control showed bending rates of 65% in the ‘Asrit Yellow’, 70% in the ‘Asrit Red’, and 20% in the ‘Merryred Pink’ cultivars (Figure 1). Surprisingly, no bending was observed in the stems of any of the cultivars treated with ethephon, even when the vase life was extended to 9days (Figures 1, 2). These results indicate an association between the bending rate and the stem curvature in these cultivars, because STS, which induced a higher stem curvature than the control, exhibited a higher bending rate than the control in all cultivars, and ethephon, which induced a relatively low curvature, did not exhibit any stem bending. In addition, the lower bending rate observed in cv. ‘Merryred Pink’ compared with the other two cultivars was also associated with the induction of a milder stem curvature in the former than the latter.

### Relative Shoot Elongation

For all cultivars, the RSE was assessed from a zone located 100mm from the apex of the flowers treated with STS, ethephon, and control for 6days. The RSE observed in the STS treatment was higher than that of the control, and the RSE observed in the ethephon treatment was the lowest (Table 1). The high, moderate, and low RSE observed in the STS, control, and ethephon treatments were associated with the bending rate (STS>control>ethephon).

**Table 1:** Comparison of relative shoot elongation of snapdragon flowers at 6 DAT with control, STS, and ethephon.

Treatment	Relative shoot elongation (% of initial)		
	cv. ‘Asrit Red’	cv. ‘Asrit Yellow’	cv. ‘Merryred Pink’
Control	21 ± 2.3 b	23 ± 2 b	16 ± 0.6 b
STS	27 ± 1.2 a	28 ± 1.5 a	20 ± 0.6 a
Ethephon	12 ± 0.6 c	10 ± 0.6 c	7 ± 0.5 c

*Data represent the mean of three replicates±standard error. Means with the same letters are not significantly different according to the least significant difference test (LSDT; p<0.05)*.

### Lignin Content and Related Gene Expression

**Figure 3:**
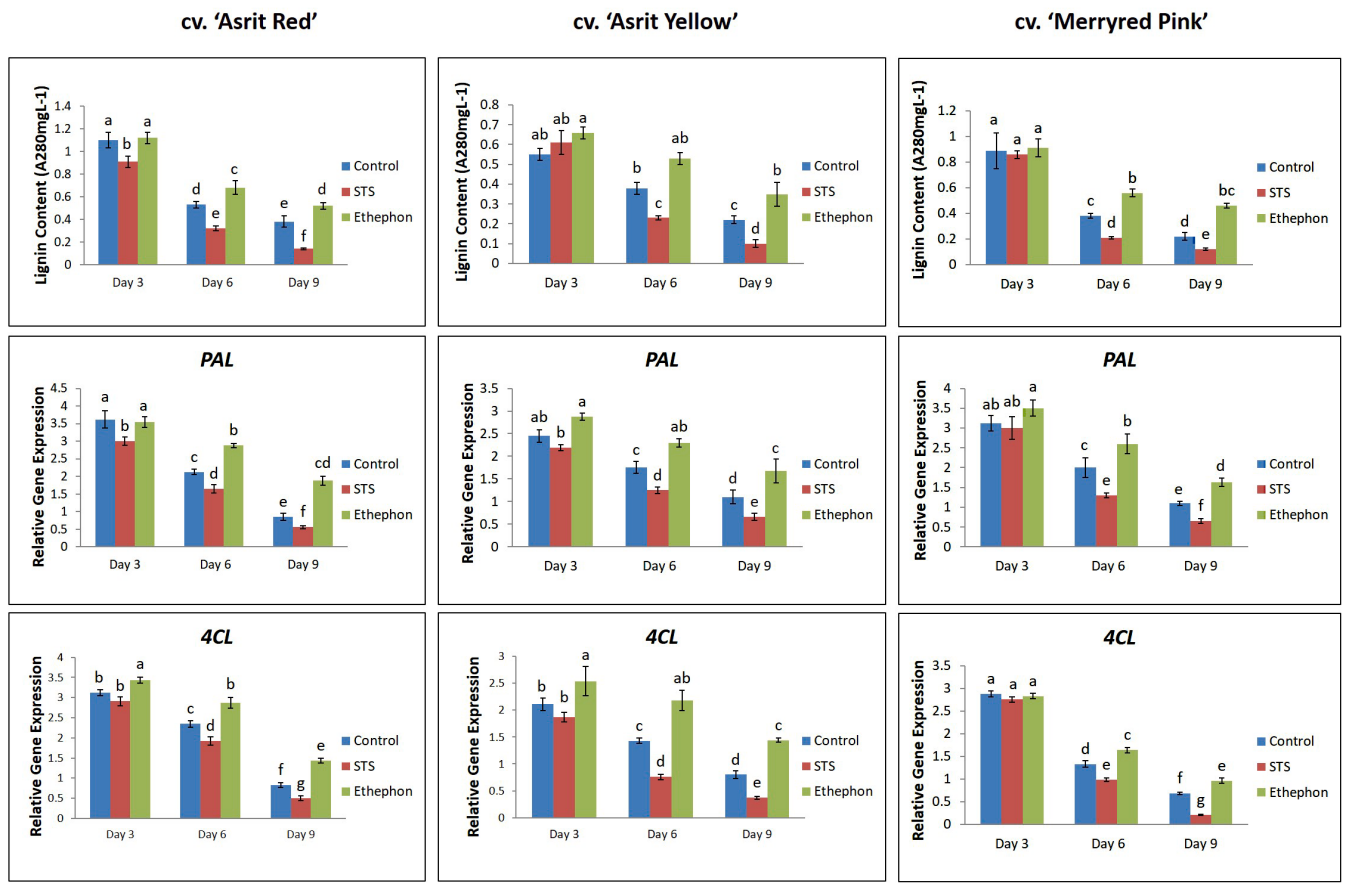
Comparison of the lignin content and related gene expression in the bending zones of stems of three different snapdragon cultivars at 3, 6, and 9 DAT with STS, ethephon, and control. The data represent the means of three replicates, and the error bars indicate the standard error. Means with the same letters are not significantly different by DMRT (*p*<0.05).

According to the results presented in Figure 1, stem bending was not observed at 3 DAT, except for the cv. ‘Asrit Yellow’ treated with STS. However, stem bending was significantly higher at 6 and 9 DAT in the STS treatment than the control, whereas no bending was observed in the stems treated with ethephon. The detection of lignin content in the bending zones of the stems at 3 DAT revealed that it was not significantly different among the treatments for all cultivars, except for the cv. ‘Asrit Red’. However, the lignin content detected at 6 DAT was significantly different among the treatments, and was significantly decreased compared with the values obtained at 3 DAT, except for the cv. ‘Asirt Yellow’ treated with ethephon. Among the treatments, the highest declination in lignin content was observed for STS, followed by the control and ethephon treatments (STS>control>ethephon). At 9 DAT, a further decrease in the lignin content was observed in all treatments for all cultivars; moreover, the values obtained for the STS and control treatments in all cultivars were significantly lower than those detected at 6 DAT, which was not observed in ethephon treatment (except for the cv. ‘Asrit Red’; Figure 3). Overall, the lignin content of cut flowers treated with STS was significantly lower than that observed for the control treatment, whereas that observed for the ethephon treatment was the highest, particularly at 6 and 9 DAT, the time points at which the stems showed bending. These results supported the occurrence of the higher bending rates in the STS than the control treatments and no bending in the ethephon treatment for all cultivars at 6 and 9 DAT. In addition, the significant decrease in lignin content in the STS and control treatments along with increasing the vase life period also supported the bending rate results (9 DAT>6 DAT>3 DAT).

The detection of the expression levels of the *PAL* and *4CL*, which are involved in lignin biosynthesis, revealed that they were generally higher at 3 DAT, followed by 6 DAT and 9 DAT, in all cultivars, indicating a continuous reduction in the expression of these genes as the vase life period increased (Figure 3). This was associated with a continuous decrease in lignin content as the vase life period increased. However, the expression levels of these genes in the ethephon treatment were significantly higher than those detected in the control and STS treatments, especially at 6 and 9 DAT. Moreover, the expression of these genes in the control was also significantly higher than that observed in the STS treatment at 6 and 9 DAT for all cultivars. These results confirmed that the lignin content in the treatments (ethephon>control>STS) was generally consistent with the expression levels of lignin biosynthetic genes (ethephon>control>STS).

### Ethylene Production and Related Gene Expression

At 6 DAT, the stem sections from the bending zones (about 100–150mm away from the apex of the shoot) of the treated flowers were excised from all cultivars, and ET production in the bending zones was assessed to determine whether its production was associated with the stem-bending rate of the cultivars. The ET production observed in the ethephon treatment was significantly higher than that observed in the control treatment, whereas the ET production detected in the STS treatment was the lowest for all cultivars (Table 2). These results suggest that there was the presence of an inverse association between ET production and stem bending in all cultivars, indicating that ET acts as a negative regulator of the snapdragon stem-bending mechanism.

**Table 2:** Comparison of ethylene production in the bending zones of stems at 6 DAT with ethephon, STS, and control.

Treatment	Ethylene production (μlkg^−1^ s^−1^)		
	cv. ‘Asrit Red’	cv. ‘Asrit Yellow’	cv. ‘Merryred Pink’
Control	2.33 ± 0.3 b	2.1 ± 2 b	2.6 ± 0.2 b
STS	0.65 ± 0.0.1 c	0.8 ± 0.2 c	1.1 ± 0.0.2 c
Ethephon	5.27 ± 0.5 a	4.5 ± 0.6 a	4.3 ± 0.5 a

*Data represent the mean of three replicates±standard error. Means with the same letters are not significantly different according to the LSDT (p<0.05)*.

**Figure 4:**
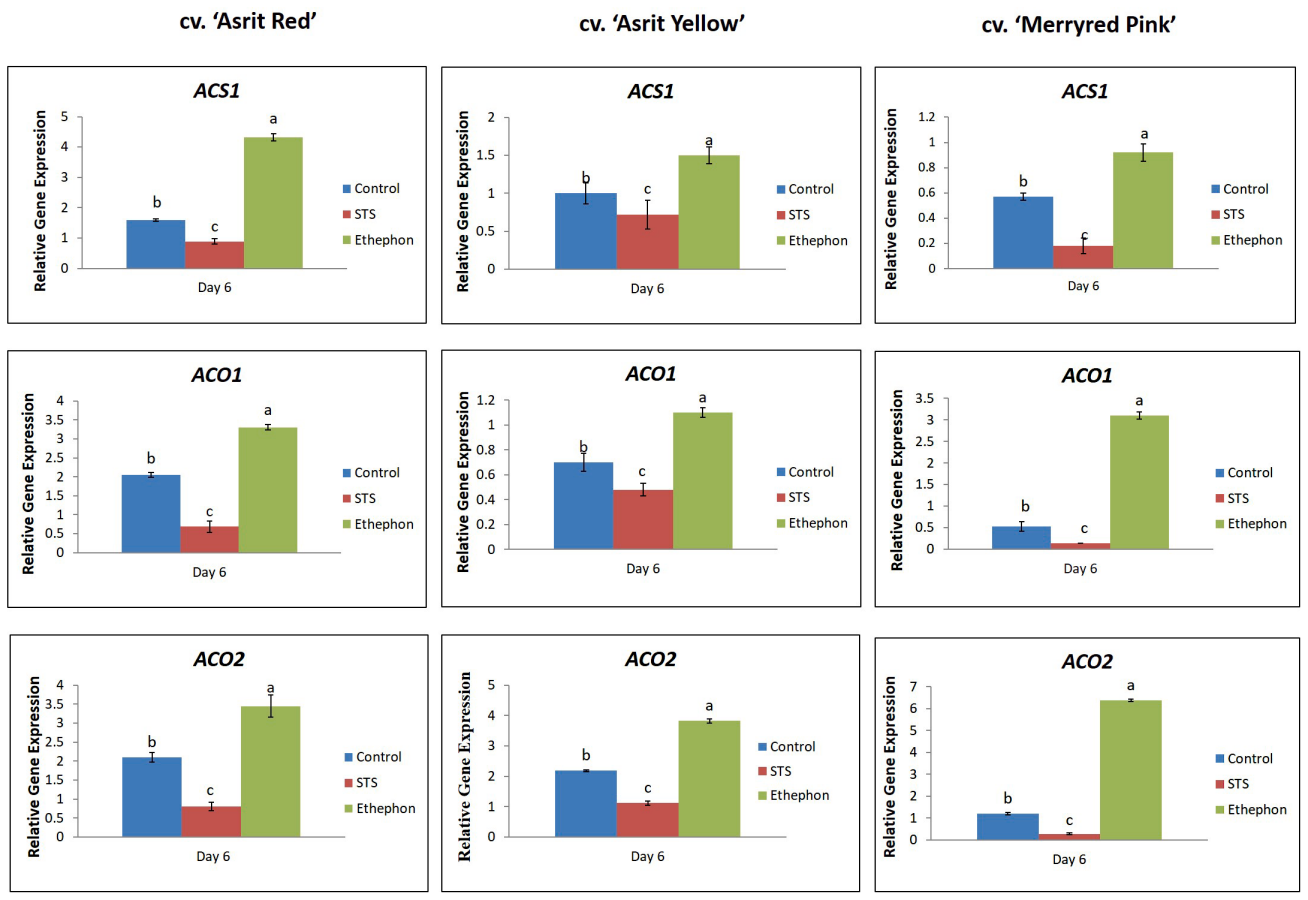
Comparison of the transcript levels of genes involved in ethylene biosynthesis in the bending zones of stems of three different snapdragon cultivars at 6 DAT with STS, ethephon, and control. The data represent the means of three replicates, and the error bars indicate the standard error. Means with the same letters are not significantly different by the LSDT (*p*<0.05).

The ET-production results were further confirmed by measuring the transcript levels of ET biosynthetic genes (*ACS1*, *ACO1*, and *ACO2*) in the stems of all cultivars. The expression levels of these genes were significantly higher in the ethephon-treated stems than the control treatment; moreover, the levels detected in STS-treated stems were the lowest for all cultivars (Figure 4). Therefore, these results indicate a positive association between ET production and the expression of its related genes in these cultivars.

## Discussion

The vase life of snapdragon cut flowers usually ends when their stems become bent. As stem bending in snapdragon flowers often occurs over a short vase life period, it has been regarded as a major problem that negatively affects the postharvest longevity of cut snapdragon flowers. ET-action inhibitors have been used to investigate whether ET acts as a positive regulator of snapdragon stem bending (Philosoph-Hadas et al., 1996, 1999; Woltering et al., 2005; Çelikel et al., 2010). However, it remains unclear how ET is involved in the stem-bending mechanism, because it has been reported as a positive regulator of stem bending in cut snapdragon flowers in some studies (Philosoph-Hadas et al., 1996, 1999), but not in different studies (Woltering et al., 2005; Çelikel et al., 2010). Surprisingly, cut tulip stems treated with ethephon, which promotes the release of ET, exhibited delayed bending (van Doorn et al., 2011). Therefore, it is interesting to investigate how ET is involved in the stem-bending mechanism of cut snapdragon flowers. In this study, we treated the cut stems of three different snapdragon cultivars with distilled water (as a control), an ET-action inhibitor (STS), and an ET-releasing compound (ethephon) and investigated how ET is involved (as a positive or negative regulator) in the stem-bending mechanism of the snapdragon cultivars by assessing stem curvature degree, stem-bending rate, and relative shoot elongation, as well as lignin content and ET production, and their related gene expression.

For all cultivars, the stem curvature degrees observed at 3 DAT were relatively low in all treatments, which indicates almost no stem bending in all cultivars at this time point. However, at 6 DAT, the degrees of curvature distinctly increased in the stems treated with STS and the control, with higher values in the STS than control treatments. These increments significantly caused increases in the stem-bending rates (STS>control) in accordance with the curvature degrees in all cultivars. In fact, the curvature degree of the stems treated with ethephon was not significantly different between 3 DAT and 6 DAT, except for the cv. ‘Asrit Yellow’, and the significant increment in the cv. ‘Asrit Yellow’ was not comparable with those observed in the control and STS treatments. This would be a reason why bending was not observed in stems treated with ethephon. A further increase in curvature degrees was observed at 9 DAT, especially for the stems treated with STS and control (STS>control), which reflected the higher bending rates in the treatments compared with 6 DAT in all cultivars. However, the curvature degree of ethephon-treated stems was not further increased in all cultivars; thus, stem bending was also not observed in any of the cultivars up to 9 DAT. These results obviously indicate that the ET-action inhibitor STS has a greater inducing effect on stem curvature and bending than does the control, whereas the ET-releasing compound ethephon completely prevented stem bending in all cultivars. These results were not consistent with those of previous studies that reported the inhibition of stem curvature by ET-action inhibitors (Philosoph-Hadas et al., 1996, 1999). Moreover, they were also different from the results reported by Çelikel et al. (2010), who suggested that ET-action inhibitors (STS or 1-MCP) do not significantly affect the gravitropic curvature and bending of snapdragon flowers. In fact, the cultivars used in the previous studies and our study vary. However, it is still difficult to claim that the discrepancy between the previous studies and our study is owing to the use of different cultivars because ethephon completely prevented stem bending of the three different cultivars used in our study. Therefore, the reason for the discrepancy between the previous studies and our study remains unknown. Among the cultivars, there was a slight variation in stem curvature and bending rates in response to the treatments, with higher curvature and bending rates in the cvs ‘Asrit Red’ and ‘Asrit Yellow’ than the cv. ‘Merryred Pink’. This might be because of the differences in the genetic and physical nature of the stems among the cultivars. Overall, preventive role of ethephon in stem-bending mechanism did not vary for all cultivars. Therefore, we concluded that ET was involved as a negative regulator of the stem-bending mechanism in the snapdragon cultivars.

At 6 DAT, the bending rates were significantly higher in the treatments (STS and controls) for all cultivars compared with 3 DAT, whereas no bending was observed in the ethephon-treated stems. The differences in stem-bending rates among the treatments were associated with the RSE of the flowers, because the RSE observed for the STS treatment was relatively higher than that of the control in all cultivars, with that observed in the ethephon treatment being the lowest. Based on these results, it was obvious that the prevention of stem bending by ethephon was due to its strong inhibitory effect on RSE and that RSE seems to be involved in the stem-bending mechanism. Previous studies have also reported a similar role of ethephon in the relative shoot growth of cut flowers (Nichols, 1971; Saniewski and de Munk, 1981; Okubo and Uemoto, 1986; van Doorn et al., 2011; Miller et al., 2012). In addition, the ethephon-induced delay in stem bending reported in the previous study may be due to its inhibitory effect on the RSE (van Doorn et al., 2011).

At 6 and 9 DAT, the bending rates were always higher in STS-treated stems than the control, and stems treated with ethephon did not show bending, for all cultivars. This was associated with the lignin content in the bending zones of the stems, because the lignin content detected in the ethephon-treated stems was significantly higher than those observed in the control, with the lowest content being observed in STS-treated stems for all cultivars. In addition, the absence of bending at 3 DAT was associated with the presence of a high lignin content in the stems of all cultivars, and the continuous increase in bending rates as the vase life period increased was also linked to the continuous decrease in lignin content. The results pertaining to lignin content were associated with the expression levels of lignin biosynthetic genes (*PAL* and *4CL*), because their expression in ethephon-treated stems was higher than that observed in the control, with the lowest expression levels being observed in the STS treatment. In addition, their expression levels were continuously lower in all treatments along with increasing the vase life in all cultivars. The occurrence of stem bending caused by a low lignin content may be explained by the fact that a low content of this molecule might not provide sufficient rigidity to the stem and vascular tissue, which would result in poor mechanical resistance to the whole-flower spikes and disruption of water and mineral transport to the flowers (Steinitz, 1982, 1983), thereby leading to rapid stem bending. A positive association between stem bending and low lignin content was reported in previous studies (Steinitz, 1982, 1983; Marousky, 1986; Perik et al., 2014; Naing et al., 2017). In this study, the presence of a higher lignin content in the ethephon treatment compared with other treatments may be due to the higher accumulation of ET in the former, because ET has been reported to promote lignification by triggering lignin biosynthetic genes (*PAL* and *4CL*) and to improve stem strength and resistance, to delay stem bending (Prasad and Cline, 1987; Brown, 1990; Hyodo et al., 1991; Ievinsh and Romanovskaya, 1991; Sitbon et al., 1999; Luo et al., 2007; Huang et al., 2013; Mangieri et al., 2016; Ye et al., 2016). The detection of ET production in the bending zones at 6 DAT revealed that, as expected, the ET production in the ethephon treatment was higher than that detected in the control, and that the ET accumulated in the STS treatment was the lowest for all cultivars. These results indicated the association between ET production and lignin content in the treatments. The ET production observed in the treatments (ethephon>control>STS) was also linked to the results of the ET biosynthesis gene expression levels (ethephon>control>STS). It appears that STS not only blocks the ET action by binding the ET-receptor sites but also inhibits endogenous ET production *via* inhibition of ethylene biosynthesis genes. The inhibition of ET production by STS has been previously observed in cut *Eustoma* flower (Ichimura et al., 1998). Similar to STS, the ET-action inhibitor 1-MCP also reduced ET production in cut rose, carnation, and lotus flowers by the suppression of *ACS1*, *ACS2*, and *ACO1* (Imsabai et al., 2010; In et al., 2013; Ha et al., 2020). Recently, Wang et al. (2020) and Wongjunta et al. (2021) observed that STS inhibited ET production in lotus leaves and orchid flowers by suppressing the expression of *ACS* and the activities of ACS and ACO enzymes. Moreover, ethephon-enhanced ET production, *ACS* and *ACO* expression, and ACS and ACO activities have also been reported in previous studies (Imsabai et al., 2010; Lee et al., 2021; Nascimento et al., 2021; Wongjunta et al., 2021). Taken together, these findings showed that ethephon completely prevented stem bending in the three different cultivars of snapdragon cut flowers and that STS significantly stimulated stem bending. Generally, stem bending was linked to a high degree of curvature and RSE, as well as lower ET production and lignin content. Based on these results, it can be concluded that ET acted as a negative regulator of the stem-bending mechanism in the three different cultivars. As similar findings were obtained in the three different cultivars, the results described in this study would be valid and will contribute to a better understanding of the role of ET in the stem-bending mechanism of other ornamental cut flowers.

## Conclusion

The mechanism that underlies the role of ET in stem bending in different snapdragon cultivars has been revealed by treating the cut stems with ethephon, STS, and distilled water (controls). Ethephon completely prevented stem bending throughout the vase life period in the three cultivars investigated, whereas STS caused stem bending faster than did the controls. Stem bending was affected by the factors such as the degree of stem curvature, RSE, and ET and lignin content. The expression analysis of genes (*PAL*, *4CL*, *ACS1*, *ACO1*, and *ACO2*) involved in lignin and ET production supported the involvement of ET and lignin in the stem-bending mechanism. Overall, ET seems to be involved as a negative regulator of stem bending in the snapdragon cultivars, and the information will be valuable for the prevention of stem bending in other commercially important ornamental flowers.

## Data Availability Statement

The datasets presented in this study can be found in online repositories. The names of the repository/repositories and accession number(s) can be found in the article/Supplementary Material.

## Author Contributions

AN designed the study and wrote and revised the manuscript. MS conducted the experiments. JY assisted in the experiments. CK supervised the project. All authors read and approved the final manuscript.

## Funding

This work was supported by the National Research Foundation of Korea (NRF) grant funded by the Korea government (MSIT) (No. 2021R1A2C2008951).

## Conflict of Interest

The authors declare that the research was conducted in the absence of any commercial or financial relationships that could be construed as a potential conflict of interest.

## Publisher’s Note

All claims expressed in this article are solely those of the authors and do not necessarily represent those of their affiliated organizations, or those of the publisher, the editors and the reviewers. Any product that may be evaluated in this article, or claim that may be made by its manufacturer, is not guaranteed or endorsed by the publisher.
