# Supplementary material for: Ethylene Acts as a Negative Regulator of the Stem-Bending Mechanism of Different Cut Snapdragon Cultivars
Source: Front Plant Sci. 2021 Sep 30;12:745038. doi: 10.3389/fpls.2021.745038 (PMC8552118; doi:10.3389/fpls.2021.745038)
Supplement: Supplementary file 1 [file Table_1.DOCX]

**Supplementary Table 1**. Primer sequences used for the detection of genes related to ethylene biosynthesis and lignin biosynthesis by qRT-PCR

| Name of Gene | Primer sequence (5’-3’) | PCR condition |
| --- | --- | --- |
| *AmACO1* | F- TGGCACTAAGGTCAGCAACT  R- ACCCATTCACCGTCCTTGAG | 95°C (10 min) 🡪 [95°C (15 s) 🡪 58.3°C (1 min) 🡪 72°C (35 s)] x 40 cycles 🡪 95°C (15 s) 🡪 58.3 °C (1 min) 🡪 95 °C |
| *AmACO2* | F- AGCACCAGCATTGGTCGAAA  R- TCTTGGCTCTTTTGCCTGGA | 95°C (10 min) 🡪 [95°C (15 s) 🡪 57.3°C (1 min) 🡪 72°C (35 s)] x 40 cycles 🡪 95°C (15 s) 🡪 57.3 °C (1 min) 🡪 95 °C |
| *AmACS1* | F- GGGCTTTACTGCTGGATGGA  R- CGAGCAATGGAACGATGCAC | 95°C (10 min) 🡪 [95°C (15 s) 🡪 59.3°C (1 min) 🡪 72°C (35 s)] x 40 cycles 🡪 95°C (15 s) 🡪 59.3 °C (1 min) 🡪 95 °C |
| *AmPAL* | F- TGCCAAAAGCAGACACGAAC  R- TAGACGTGGCCGAGTGAAAC | 95°C (10 min) 🡪 [95°C (15 s) 🡪 58.3°C (1 min) 🡪 72°C (35 s)] x 40 cycles 🡪 95°C (15 s) 🡪 58.3 °C (1 min) 🡪 95 °C |
| *Am4CL* | F- TGTCACCTGTTGCTGAACCA  R- AGGCTGAGGGATGCACTCTA | 95°C (10 min) 🡪 [95°C (15 s) 🡪 58.3°C (1 min) 🡪 72°C (35 s)] x 40 cycles 🡪 95°C (15 s) 🡪 58.3 °C (1 min) 🡪 95 °C |
| *AmACT1* | F- CAAGAAATGGAGACGGCCAAG  R- TCCAGCAGATTCCATTCCGA | 95°C (10 min) 🡪 [95°C (15 s) 🡪 57°C (1 min) 🡪 72°C (35 s)] x 40 cycles 🡪 95°C (15 s) 🡪 58.3°C (1 min) 🡪 95 °C |
